# The Colored Population—Mulattoes, Etc.

**Published:** 1863-10

**Authors:** Sam’l G. Howe

**Affiliations:** 143 Second Ave., Cor. East Ninth St., N. Y.


### THE COLORED POPULATION—MULATTOES, ETC.

[COMMUNICATED.]

A Commission has been appointed by the Government to enquire into the condition of the Colored population emancipated by the President's Proclamation and by the Act of Congress, and to report what measures are necessary to place them in a condition of self-support and self-defence, with the least disturbance to the great industrial interests of the country.

This Commission are seeking to ascertain the vital statistics concerning the African race, and the Mulattoes, as well in the Northern and Middle as in the Southern States. It is very important that this should be done, but unfortunately the data do not exist. It is not known, from any wide circle of observation, whether the Mulattoes are as fertile as Blacks and Whites; whether they are long lived; nor even whether their breed can exist permanently; that is, whether its hybridity will prevent its persistence.

Then there are questions about the adaptation of the cross breed to the Northern parts of the temperate zone; questions about the effects of amalgamation upon the white race and the like. The Commission have sent out circulars to many Medical men, but of course will not reach all who might, if called upon, give valuable aid. We therefore print the series of questions put forth by Dr. Howe, in behalf of the Commission, and invite the attention of our readers to it.

1. What is the number of the colored population of your town?

2. About how many pure blacks?

3. " " " mulattoes?

4. Does the colored population, if not recruited by immigration, increase or decrease?

5. Do mulattoes seem to you to have as much vital force to resist disease and destructive agencies as pure blacks, and as whites; and they usually live as long?

6. To what diseases do mulattoes seem peculiarly liable?

7. Do mulatto families usually have as many children as white families?

8. Can you give instances, within your own knowledge, of the number of children in one family born of, and reared to maturity by, mulatto parents?

9. Are the colored people generally industrious and self-supporting, or not?

10. How is it in the second generation with regard to the number and health of offspring?

11. Through how many generations has any family of mulattoes been known to persist?

12. Do the mulattoes seek public charity in greater or less proportion than whites?

13. Do you consider them, upon the whole, as valuable members of the community, or not?

Those who are disposed to answer the queries, or to favor the Commission with their views upon the general subject committed to it, are invited to address,

Dr. SAM'L G. HOWE, 143 Second Ave.,
Cor. East Ninth st., N. Y.

---

We are in receipt of Lindsay & Blakiston's Physician's Visiting List, Day-Book, and Memoranda for 1864. The convenience of this little work is so well known as to require no comment. The publishers send it by mail, free of postage, on the receipt of the retail price, viz.: for 25 patients, cloth, 62 cents, leather with tucks and pencil, $1; for 50 patients, cloth 75 cents, leather, $1.25; for 100 patients, $2; interleaved, for 25 patients, from 75 cts. to $1.25; for 50 patients, $1 to $1.50.

*Ovariotomy in France.*—M. Hugnier lately operated in Paris upon an English girl, aged twenty, whose abdominal development was equal to that observed at the term of gestation. She was operated upon at Bellevue, in the immediate neighborhood of Paris, in a house hired by the hospital authorities especially for the purpose of giving operations of this kind every chance of success. Before reaching the ovarian cyst the operator came upon a nodulated mass, which the gentlemen surrounding him at first took for intestine. It was, however, a cyst developed in the omentum, and which lay between the ovarian cyst and the abdominal walls. M. Hugnier considers that no complication of this kind was ever recorded. The ovarian tumour being reached, was found to consist of some hundreds of compartments, some of which were filled with a thick ropy fluid, others with a clear and transparent liquid. The pedicle was seized and tied with a silver wire, and cut off clean. The omentum, which contained about a dozen little cysts, was tied in the same manner and cut off. The operation lasted nearly an hour, and the patient died about forty-eight hours afterwards of peritonitis.—*London Lancet.*

---

*Anecdote of Baron Larrey.*—In a charge of British cavalry at Waterloo, Colonel Weymouth, whether impelled by his own bravery or by some other cause, did not stop till he found himself amidst some French cuirassiers. He then endeavored to ride back, but he was pulled off his horse and wounded. One Frenchman levelled his gun at him, and was taking deliberate aim, when a French officer humanely knocked the gun down with his sword. He then addressed Weymouth in broken English and in a kind tone of voice. He also took a penknife out of his pocket, and said, "Look at thees canif, what you call penknife—Cockspur street, Londres. I was in your country." It was Baron Larry. The British officer, who had previously been fighting under the Duke of Wellington in the Peninsular War, would have entered the next world on the 18th of June, 1815, if the Surgeon-in-Chief of the French army had not interposed to save his life. He is at present residing in this land of good cutlery, deservedly esteemed by a large circle of friends.—*Ibid.*

---

☞ Business letters to the Journal should be addressed to Dr. E. INGALS, Chicago, Ills., P. O. Drawer 5787. When money is received a receipt will be returned with the next number of the Journal, and subscribers failing to get such receipt will confer a favor by giving us notice of the omission.

